# Development and Validation of a Scale to Measure Job Satisfaction Among Human Resources for Health in Morocco

**DOI:** 10.7759/cureus.57438

**Published:** 2024-04-02

**Authors:** Wafâa Al Hassani, Youness El Achhab, Mohammed Taiebine, Chakib Nejjari

**Affiliations:** 1 Euromed Research Center, Euromed University of Fez, Fez, MAR; 2 Biological Sciences, Regional Center for Education and Training Careers of Fez-Meknes, Fez, MAR

**Keywords:** job satisfaction, human resources for health, validity, reliability, scale development

## Abstract

This study aims to develop and validate a job satisfaction scale for human resources for health (HRH) who are employed by the Ministry of Health. The scale was developed through a comprehensive literature review, and its validity and reliability were assessed using several psychometric properties, including expert evaluation, a pilot survey, exploratory factor analysis (EFA), and confirmatory factor analysis (CFA). A large sample psychometric evaluation was made by all kinds of HRH staff (n = 2122), and the final version of the job satisfaction scale included 25 items. The EFA revealed seven factors with modest internal consistency ranging from 0.68 to 0.85. The goodness of fit of the model was found to be satisfactory, with root mean square error approximation (RMSEA) = 0.05, chi-square/df = 6.4, and both Tucker-Lewis Index (TLI) = 0.92 and CFI = 0.93 being higher than 0.9. The standardized root mean square residual had a value of 0.035. This instrument proved to be a reliable and valid tool for measuring job satisfaction in health institutions.

## Introduction

Job satisfaction is understood as an individual's perception of their job [1]. Alternatively, it is described as the feelings individuals have towards their jobs and their various facets, encompassing the degree to which individuals enjoy (satisfaction) or are displeased with (dissatisfaction) their work [2]. Additionally, it is noted that a worker who is satisfied with their job tends to experience a higher level of engagement in their work [3]. Several components of job satisfaction were mentioned in several meta-analyses and systematic reviews, namely, working conditions, the work itself, workload, the institution's relationship, organizational culture, remuneration, opportunities for advancement, psychological rewards, job security, and leadership styles [4-6].

Job satisfaction plays a crucial role in boosting employees' motivation and productivity [7,8]. It acts as a vital indicator, allowing senior management and policymakers to continuously assess achievement levels within the job scope. This assessment is essential for exploring diverse strategies to enhance job management and enrichment. Without diligent tracking of job satisfaction, employees' behaviors might negatively influence their work atmosphere, subsequently diminishing their output [9]. While the job satisfaction questionnaire is often viewed as a standard tool for study, it is imperative to periodically re-evaluate the specific domains and items it measures. This reassessment is particularly crucial given the array of contemporary challenges confronting employees in their workplaces, notably within the healthcare sector.

Therefore, this newly created questionnaire will be invaluable in offering ongoing feedback to healthcare policymakers and managers within medical institutions regarding the levels of job satisfaction among the healthcare workforce, periodically. This approach will aid in addressing any unfavorable working conditions that arise as contributing factors to job dissatisfaction among employees. Therefore, this study aims to develop and validate a job satisfaction scale for the healthcare workforce that is employed by the Ministry of Health in Morocco.

## Materials and methods

Study design and participants

The study population refers to all the health professionals working in Morocco. A sample of study respondents was recruited by adopting a stratified two-stage survey in the year 2018. In the first stage, 160 institutions were included. To ensure accurate representation based on healthcare categories, individual selection was conducted through a tailored random selection grid for each chosen establishment, adhering to the distribution of categories within each establishment. Data were collected through face-to-face interviews.

The research protocol was approved by the Ministry of Health, which granted permission to conduct the study at the national level. Next, we asked for the voluntary participation of all participants, providing them with written information on the aims of this study as well as on the protection of their anonymity.

Process of questionnaire development

A comprehensive literature search was conducted in PubMed and Google Scholar to identify existing tools that measured the job satisfaction of health professionals. Three main instruments were identified, which were as follows: The Saphora-Job Questionnaire [10], which is designed as a "general" scale that has been tailored to the healthcare sector's unique needs which stands out for its relevance as a versatile tool adapted to the healthcare sector's specificities, targeting all sector employees, not limited to care professionals or healthcare managers; the Minnesota Satisfaction Questionnaire, which was developed to measure the individual’s satisfaction with 20 different aspects [11]; and the Job Descriptive Index, which measures five factors [12].

These instruments were adopted for use in this study based on the alignment of the majority of their attributes or items with the dimensions and areas of analysis prioritized by the project team. In the first phase, 16 sub-areas were identified. On the basis of the literature review and expert panel consultation, 49 items related to job satisfaction were identified. These members of the expert panel were working with or closely collaborating with the Ministry of Health. The content analysis of these items revealed 13 sub-areas of job satisfaction. Members of the research group also reviewed and made changes to the items on a number of occasions, including after a pilot test involving 30 health professionals.

The number of items was subsequently reduced to 33, following the removal of sub-areas more closely related to motivation than job satisfaction. A test item was created for each of the 33 items, and respondents were asked to answer each item on the test using a five-point Likert scale.

Statistical analysis

Initially, descriptive statistics were used to provide an overview of the socio-demographic characteristics of all study participants. Categorical variables were presented in terms of both number and frequency. The relationships between categorical variables were examined through contingency tables and by calculating the chi-squared test. P-values were based on two-sided tests and compared to a significance level of 5%.

Exploratory factor analysis (EFA) and confirmatory factor analysis (CFA) were employed to assess construct validity, ultimately guiding the identification of the optimal construct within the scale for evaluating job satisfaction levels.

In the first phase, EFA was used to help reduce the number of items on the scale and identify any underlying latent variables. A sample of 1,500 health professionals’ data was used to perform this analysis. Principal axis factoring with varimax rotation was employed due to the anticipation of a theoretical underlying factor structure informed by the findings of the systematic literature review. In cases of cross-loading or loading of less than 0.40, items were deleted. In the second phase, the model fit was then assessed on 2,122 health professionals’ data by using CFA, where indicators such as Comparative Fit Index (CFI) ≥ 0.90, Tucker-Lewis Index (TLI) ≥ 0.90, root mean square error approximation (RMSEA) ≤ 0.08, and chi-square/df <5 were estimated [12,13]. Statistical analyses were performed using Jamovi 2.3.28 software (The Jamovi project (2023). Retrieved from https://www.jamovi.org).

## Results

Table 1 represents the characteristics of the participants. A total of 2,122 study participants were included in this study, with the majority being female (n = 1,231; 58.3%). The age range of 40 to 50 years was represented by 32.6% (n = 688) of the participants. The largest proportion of these participants had more than 10 years of experience (n = 1,378; 65.3%). Nurses and midwives represented half of the participants (n = 1,070; 50.4%). The composition of participants remained consistent across the two phases of analysis, except for age distribution (p<0.05).

**Table 1:** Characteristics of participants in the first and second phases The data have been represented as N, %. The difference is considered significant when p<0.05.

	First phase (n = 1,500)		Second phase (n = 2,122)		p-value
	n	%	n	%	
Gender					0.608
Female	881	59.2	1,231	58.3	
Male	608	40.8	880	41.7	
Age (years)					0.026
Under 30	189	12.7	224	10.6	
30 to 39	440	29.6	641	30.4	
40 to 49	430	28.9	688	32.6	
50 or older	430	28.9	558	26.4	
Work experience					0.103
Less than 2 years	67	4.5	72	3.4	
2 years to less than 5 years	178	12.0	215	10.2	
5 years to less than 10 years	312	21.0	446	21.1	
10 years or more	932	62.6	1,378	65.3	
Position					0.817
Nurses and midwives	726	48.4	1,070	50.4	
Specialist and generalist	302	20.1	416	19.6	
Administration staff	121	8.1	159	7.5	
Technical staff	331	22.0	450	21.2	
Others	20	1.3	27	1.3	

Following the execution of EFA employing principal axis factoring with varimax rotation, which identified factors with parallel analysis, the designed questionnaire was structured to include 25 items distributed across seven domains (Appendix A), namely: career development (six items), working conditions (four items), social support (four items), role clarity (three items), workload (four items), remuneration (two items), and the institution's relationship (two items). Together, the seven factors explained 54.4% of the total variance.

Each item in the scale seamlessly aligned with its designated domain, both in terms of content and as determined by pertinent statistical analyses (Table 2). Across all domains of the scale, the lowest factor loading for any item stood at 0.460. The Cronbach’s alpha values for the scale ranged from 0.68 to 0.85, indicating that the scale has only a modest degree of internal consistency [14].

**Table 2:** Result of EFA and internal consistency for scale which consists of 25 items and seven domains The exploratory factor analysis (EFA) was conducted based on principal axis factoring using the varimax rotation method. Q: question; CD: career development; WC: working condition; SS: social support; RC: role clarity; WL: workload; R:  remuneration; IR: institution's relationship Loadings of less than 0.40 were not included in the table.

Items	Domains							Cronbach’s alpha
	CD	WC	SS	RC	WL	R	IR	
Q30	0.799							0.876
Q32	0.733							
Q29	0.727							
Q31	0.710							
Q33	0.643							
Q28	0.596							
Q26		0.754						0.734
Q25		0.747						
Q24		0.647						
Q27		0.490						
Q14			0.650					0.785
Q18			0.615					
Q19			0.609					
Q15			0.524					
Q5				0.853				0.789
Q4				0.727				
Q6				0.514				
Q10					0.673			0.784
Q1					0.645			
Q9					0.495			
Q2					0.460			
Q22						0.842		0.811
Q21						0.803		
Q12							0.809	0.766
Q13							0.738	

To determine the fit of the structured model, which was developed using EFA, it was later re-examined using CFA (Table 3). The chi-square/df was 6.460, which is slightly superior to 5. Several indicators of the goodness of fit of the model were found to be satisfactory, with RMSEA = 0.05; both TLI = 0.92 and CFI = 0.93 which were higher than 0.9. Finally, the standardized root mean square residual (SRMR), which measures the average magnitude of the discrepancies between observed and expected correlations as an absolute measure of fit criterion, had a value of 0.035. A value <0.10, or even 0.08, indicated a good fit [15].

**Table 3:** Model fit indices

Model fit indices	References	Values
Chi-2/df	<5	6.460
Tucker-Lewis Index (TLI)	≥ 0.90	0.92
Comparative Fit Index (CFI)	≥ 0.90	0.93
Root mean square error approximation (RMSEA)	≤ 0.08	0.05
Standardized root mean square residual (SRMR)	≤ 0.08	0.035

## Discussion

The aim of this research was to develop a job satisfaction scale applicable to all human resources for health (HRH). Despite the existence of numerous studies on job satisfaction and the development of various scales over recent decades [16,17], the creation of a job satisfaction scale for the national project on health professionals’ satisfaction has proven beneficial. It provides reliable measurements and results, facilitating further research and development efforts.

The validity of this scale was assessed through several approaches, such as expert evaluation, a pilot study, and exploratory and confirmatory factor analysis. These methods demonstrated the scale's validity, with its structure and the coherence of its domains being confirmed in terms of content. Additionally, Cronbach's alpha values affirmed the instrument's internal consistency, underscoring its reliability. The final construct of the scale developed in this study has now been designed to consist of a total of seven domains with 25 items. The seven domains are career development, working conditions, social support, role clarity, workload, remuneration, and the institution's relationship.

Career development is a crucial practice that boosts employee engagement, which, in turn, significantly enhances organizational effectiveness [18]. Previous studies conclude that opportunities for career development, working time, and promotional schemes of organizations have high associations with job satisfaction [18-21]. Similarly, other research has discovered that career development and compensation significantly affect organizational commitment via job satisfaction [22,23]. Certain factors play pivotal roles in career development, notably the leadership's involvement and the provision of feedback, both of which are facilitated by the human resources department. Health decision-makers can implement various career development programs, including training and education, compensation system adjustments, promotion initiatives, and group learning opportunities [18].

Currently, various organizations and institutions are facing challenges related to the working environment. Job satisfaction is significantly impacted by the conditions of the workplace. The work environment plays a crucial role in shaping an individual's sense of self-pride and satisfaction with the work they perform. It is recognized that working conditions significantly impact job satisfaction, as they directly affect the quality of the physical environment in which individuals work [24]. Consequently, 'working conditions' encompass various elements of the workplace, including sufficient workspace, the presence of office equipment, security space, low noise levels, comfortable temperature, access to necessary utilities like electricity and water, and space hygiene and cleanliness.

Existing literature presents a multitude of determinants of job satisfaction, showcasing the wide range of factors associated with the topic. Social support, role clarity, workload, remuneration, and the institution's relationship have been reported in numerous studies [18,25-27]. In a broad sense, this alignment pertains to the level of congruence between an employee's values, beliefs, interests, and needs and the workplace's values, norms, and culture.

The current body of literature on the various aspects of job satisfaction unanimously supports the concept of the predicted 7 domains in 25 items of the job satisfaction scale. Furthermore, the validity and reliability of the scale were substantiated through both the EFA and the CFA. Moreover, if the internal consistency of each domain within the construct is determined to be suitably high and the model's fit is deemed satisfactory by CFA, then the scale's construct can be regarded as a reliable and valid tool for assessing job satisfaction.

This research enlisted a total of 2,122 participants, clearly greater than the minimum sample size needed for both EFA and CFA. Another major achievement of this study for the validation of this scale is that it has been validated among all kinds of healthcare workers. However, a significant limitation of this study, of which the authors are fully aware, is the exclusion of certain aspects of 'job satisfaction' that could impact the subject. Factors such as training [18], lifelong learning, emotional intelligence, and leadership [26], which have been associated with the concept of 'motivation" [28-30], were not included.

## Conclusions

The scale has been determined to be a valid and reliable tool for assessing job satisfaction among healthcare workers. It is also suitable for various other applications, including management and research projects that require an evaluation of job satisfaction. Integrating 'job satisfaction' and 'motivation' into a single instrument could effectively address the diverse perspectives related to situational factors impacting job satisfaction, as well as the consistency and dynamics of responses to work conditions.

## Appendices

Appendix A

Job Satisfaction Scale

Please take your organization into consideration as you respond to each statement. Kindly read each statement carefully and determine the degree to which you agree with the statements below. Please mark only one answer for each statement.

**Table 4:** Job satisfaction scale

Part 1			
Gender	Age (years)	Experience (years)	Function
Institution	Type of Institution	Service (department)	Locality (city)

Part 2					
Q1	How do you evaluate your workload?				
	Overloaded	Loaded	Accurate	Lightly loaded	Very lightly loaded

		Strongly disagree	Disagree	Uncertain	Agree	Strongly agree
Q2	The facility is adequately equipped, both in terms of resources and capacity, to meet the demands of care service users.					
Q4	Within our department, roles and responsibilities are effectively distributed.					
Q5	Within our institution, roles and responsibilities are effectively distributed.					
Q6	The goals and priorities of your work are explicitly stated.					

Q9	How satisfied are you with your current job?				
	Very unsatisfied	Unsatisfied	Accurate	Satisfied	Very satisfied
Q10	How much stress does your job cause you?				
	Very stressful	Stressful	Accurate	Little stressful	Not stressful

		Strongly disagree	Disagree	Uncertain	Agree	Strongly agree
Q12	Professional relationships within your department are cordial and respectful.					
Q13	You receive assistance from co-workers when necessary.					

	How would you rate:	Very Unsatisfying	Unsatisfying	Accurate	Satisfying	Very satisfying
Q14	The comfort of the institution (heating, ventilation, noise levels, lighting, and space)?					
Q15	Security around the institution's premises					
Q18	Hygiene and cleanliness of spaces					
Q19	Safety in medical procedures					

	Do you believe the remuneration system should shift towards a variable model that reflects an:	Strongly disagree	Disagree	Uncertain	Agree	Strongly agree
Q21	employee's performance?					
Q22	employee's attendance?					

	How do you rate the ministry's social offerings in terms of:	Very Unsatisfying	Unsatisfying	Accurate	Satisfying	Very satisfying
Q24	Pensions and medical coverage					
Q25	Occupational accidents					
Q26	Benefits for children					
Q27	Other benefits (cultural, sporting, pilgrimage )					

	How do you rate:	Strongly disagree	Disagree	Uncertain	Agree	Strongly agree
Q28	The evaluation system is equitable and acknowledges the worth of your contributions.					
Q29	Your progress is consistent with your achievements.					
Q30	The process for career development and management is dynamic, providing opportunities for career advancement.					
Q31	The process for promotions and career management is conducted with transparency.					
Q32	The policy on assignments and transfers is conducted with transparency.					
Q33	The Ministry's promotion policy is motivating.					
